# Regenerative therapies for complex diabetic foot wounds: a therapeutic challenge

**DOI:** 10.1590/1677-5449.202500712

**Published:** 2026-04-10

**Authors:** Vinicius Tadeu Ramos da Silva Grillo, Eduardo Aparecido Romio, Caíque Damasceno Sousa, Gabriel Gomes Ranite, William Rodrigues da Silva, Pedro Luciano Melluci, Francisco José de Oliveira, Matheus Bertanha

**Affiliations:** 1 Universidade Estadual Paulista – UNESP, Faculdade de Medicina de Botucatu – FMB, Botucatu, SP, Brasil.; 2 Centro Universitário São Lucas – UNISL, Porto Velho, RO, Brasil.; 3 Instituto Vascular e Endovascular de Rondônia – IVER, Porto Velho, RO, Brasil.

**Keywords:** diabetic foot, wound healing, negative-pressure wound therapy, extracellular matrix, mesenchymal stem cells, chronic limb-threatening ischemia

## Abstract

Diabetic foot ulcers are a leading cause of morbidity, hospitalization, and nontraumatic lower-extremity amputation in patients with diabetes mellitus. Effective management of these wounds requires a multidisciplinary approach and combined therapies addressing infection control, perfusion optimization, and stimulation of tissue regeneration. This case report describes a patient with a severe infected neuroischemic foot ulcer treated with a sequential integrated strategy: infrapatellar endovascular revascularization, targeted antibiotic therapy, serial debridement, negative pressure wound therapy, application of an ovine-derived extracellular matrix, and autologous grafting of adipose tissue-derived mesenchymal stem cells. The treatment resulted in complete wound healing and limb preservation, with no recurrence after 7 months of follow-up. This case highlights the potential role of advanced regenerative therapies in the management of complex, refractory diabetic foot wounds, expanding therapeutic perspectives in the care of patients with diabetic foot.

## INTRODUCTION

Diabetes mellitus is one of the leading causes of morbidity and mortality worldwide. According to the IDF Diabetes Atlas 11th edition 2025, Brazil ranks sixth globally in disease prevalence, with approximately 16.6 million adults diagnosed.^1^ Among its most serious and costly complications is diabetic foot, a condition resulting from the interaction of anatomical, orthopedic, vascular, neurological, and infectious abnormalities.^2,3^ Diabetic foot significantly impairs patients’ quality of life and remains the leading cause of nontraumatic lower-extremity amputations.^4^

Complex diabetic foot wounds may be classified as neuropathic, ischemic, or neuroischemic, the latter being associated with a poorer prognosis. Multiple therapeutic strategies have been developed for the management of these wounds, including advanced wound dressings, serial debridement, autologous and heterologous grafts, negative pressure wound therapy (NPWT), mesenchymal stem cell (MSC) therapy, gene therapy, hyperbaric oxygen therapy, dermal substitutes, and topical antibiotics.^5^ These interventions are considered adjuvant therapies, without a direct impact on the pathophysiology of the disease, and have not yet been incorporated into international guidelines, which recommend caution due to the need for larger, methodologically robust studies.^6^ Furthermore, the routine use of topical antibiotics in diabetic foot ulcers is not recommended.

Despite the available treatment options, the management of neuroischemic ulcers remains particularly challenging due to impaired wound healing, recurrent infection risk, and a high likelihood of major amputation. Multimodal strategies combining revascularization, strict infection control, and emerging regenerative therapies have gained attention as promising approaches in the current scenario.^7,8^

The present case report aims to describe the integrated application of regenerative therapies in a patient with a complex ischemic diabetic foot wound, highlighting the clinical challenges encountered and discussing the potential role of regenerative medicine in the treatment of such wounds.

This study was reviewed and approved by the Institutional Research Ethics Committee (CAAE 86657025.5.0000.0013; approval no. 7.476.797).

## PART I – CLINICAL PRESENTATION

A 65-year-old man presented to the emergency department with a 40-day history of having stepped on hot embers, subsequently developing ulcerative lesions on both feet. He had a history of type 2 diabetes mellitus and systemic arterial hypertension for more than 15 years, with irregular use of oral hypoglycemic agents and antihypertensive medications, which he had discontinued on his own in the preceding months. He reported chronic alcohol consumption and smoking cessation more than 30 years earlier. There was no history of prior surgical procedures.

On physical examination, infected necrosis of the fourth and fifth toes of the left foot was observed, with ascending lymphangitis over the dorsum of the foot. On the right foot, a small superficial plantar lesion was noted, without inflammatory signs. The anterior tibial and dorsalis pedis pulses were palpable on the right foot; on the left foot, only the popliteal pulse was detectable, with absent distal pulses and an ankle-brachial index (ABI) of 0.5. Monofilament testing was not performed. However, the medical history and physical examination indicated distal symmetrical peripheral neuropathy.

The wound was classified as Wagner grade 4.^9^ According to the Society for Vascular Surgery Wound, Ischemia, and foot Infection (WIfI) classification, the wound was graded as W3 I2 fI2, indicating an extensive wound (W3), moderate ischemia (I2), and moderate infection (fI2), suggesting a high risk of amputation without appropriate intervention.^10^

Preoperative computed tomography angiography and arteriography were not performed due to systemic infection and the need for urgent surgical intervention.

Laboratory tests revealed hemoglobin of 7.1 g/dL, leukocytosis (19,900/µL), erythrocyte sedimentation rate of 100 mm/h, C-reactive protein >160 mg/L, albumin of 2.8 g/dL, creatinine of 1.7 mg/dL, sodium of 128 mEq/L, and an international normalized ratio of 1.47.

**Figure 1:**
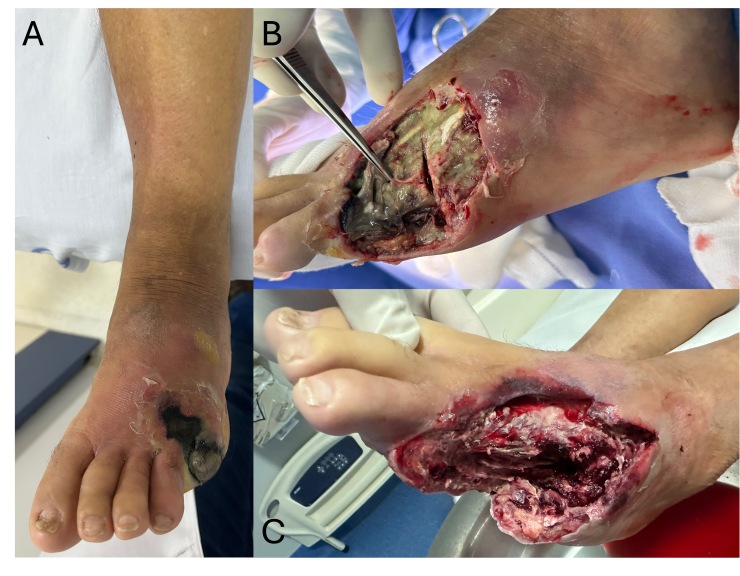
Photographs of the wound. **A)** Initial evaluation. **B)** Intraoperative view during the first intervention, after amputation of the fifth metatarsal. Note the liquefactive necrosis of the fourth metatarsal. **C)** Final appearance after the first surgical intervention, including amputation of the fourth and fifth metatarsals, plantar fasciotomy, and extensive debridement.

Empiric antibiotic therapy was initiated with ciprofloxacin (400 mg intravenously every 12 hours) and clindamycin (600 mg intravenously every 8 hours), selected for their broad spectrum against gram-negative and anaerobic organisms commonly isolated in polymicrobial diabetic foot infections. The patient also received transfusion of 3 units of packed red blood cells. Urgent surgical management was required, consisting of amputation of the left fourth and fifth toes, extensive plantar fasciotomy, and collection of material for culture, which grew multi-sensitive *Escherichia coli*. Preoperative and initial intraoperative images are shown in Figure 1, illustrating the extent of necrosis and the appearance of the wound bed after initial debridement.

## PART II – WHAT WAS DONE

**Figure 2:**
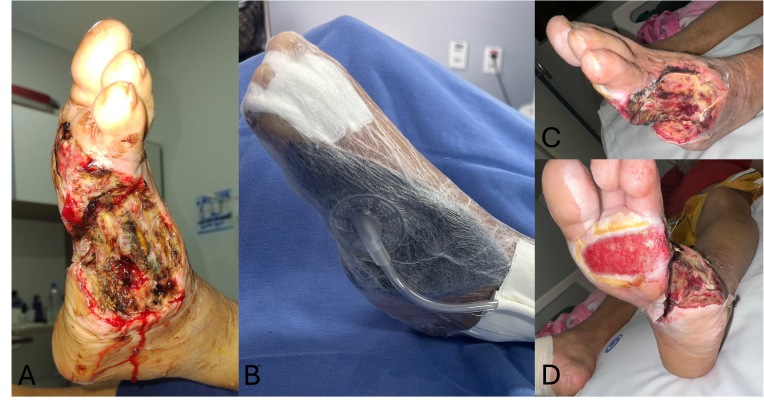
Photographs of the wound. **A)** Wound appearance after the first intervention. **B)** Dressing with negative pressure wound therapy (NPWT). **C** and **D)** Wound progression after initiation of NPWT 21 days after the first intervention.

Four days after the initial procedure, repeat surgical debridement was performed to remove devitalized tissue and optimize the wound bed, followed by initiation of NPWT at continuous −125 mm Hg (Figure 2). The decision to use NPWT was based on clinical judgment supported by evidence demonstrating its role in exudate control, bacterial burden reduction, and stimulation of granulation tissue formation.^11,12^

**Figure 3:**
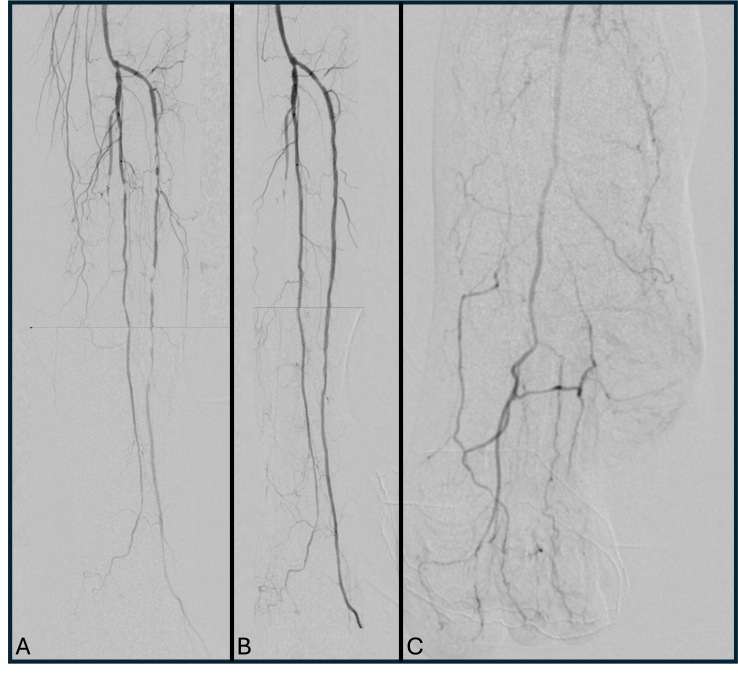
Digital subtraction arteriography images of the infrapatellar segment. **A)** Initial diagnostic arteriogram demonstrating occlusion of the posterior tibial artery and multiple stenoses and subocclusions of the peroneal and anterior tibial arteries. **B)** Final arteriogram after balloon angioplasty of the peroneal and anterior tibial arteries. **C)** Final arteriogram with anteroposterior view of the foot.

On hospital day 11, diagnostic arteriography confirmed significant obstructive lesions in the infrapatellar arteries of the left lower extremity: occlusion of the posterior tibial artery and multiple stenoses and subocclusions of the peroneal and anterior tibial arteries. Percutaneous transluminal balloon angioplasty of the infrapatellar arteries was performed, with technical success evidenced by vessel recanalization, restoration of a palpable anterior tibial pulse, and a post-procedure ABI of 0.9 (Figure 3). Subsequent pharmacological management included dual antiplatelet therapy with acetylsalicylic acid (100 mg/day) and clopidogrel (75 mg/day) for 90 days, in addition to regular high-intensity statin therapy (rosuvastatin 20 mg/day).

Antibiotic therapy was maintained for 21 days. During hospitalization, NPWT was used continuously, with dressing changes every 7 to 10 days, each accompanied by serial debridement performed in a surgical unit and documentation of granulation tissue progression. The patient was discharged on hospital day 40 and continued treatment in a day-hospital outpatient setting. A total of 11 NPWT cycles were performed, maintained uninterrupted through day 120, with regular dressing changes also in a surgical unit, each accompanied by debridement and wound reassessment. Subsequently, outpatient follow-up continued with daily hydrogel dressings until complete wound healing.

**Figure 4:**
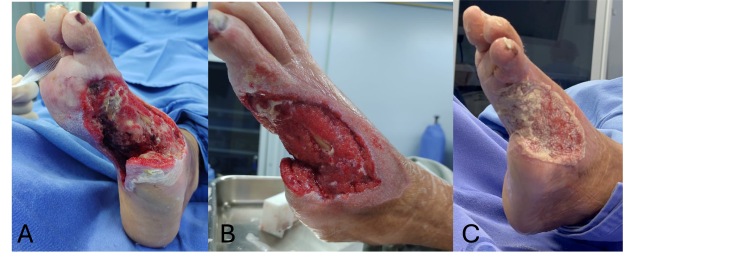
Photographs of wound progression. **A** and **B)** Forty days after the first intervention. **C)** Sixty days after the first intervention; note the placement of a dermal matrix in the wound bed.

An extracellular matrix (ECM) derived from ovine small intestinal submucosa (Endoform®) was indicated due to the presence of immature granulation tissue and atrophic wound edges, with the aim of promoting angiogenesis and endogenous collagen deposition. Progress was monitored through photographic documentation and serial wound area measurements (Figure 4).

**Figure 5:**
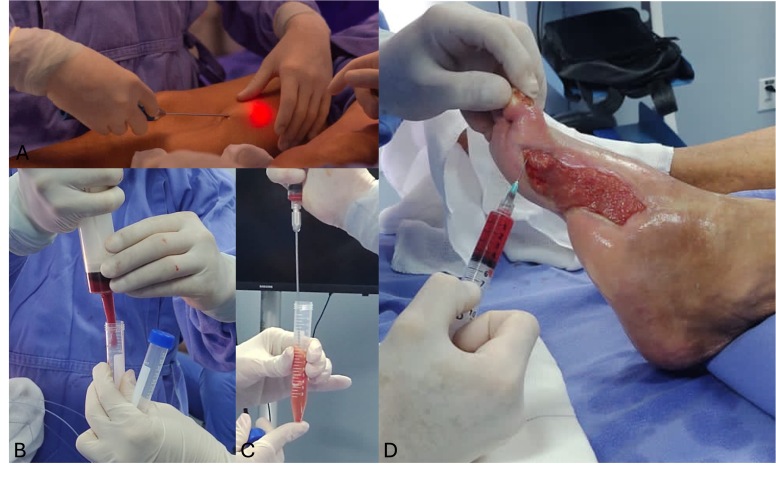
Photographs of the mesenchymal stem cell (MSC) grafting procedure performed 3 months after the first intervention. **A)** Liposuction of adipose tissue from the thigh. **B)** Transfer of harvested material into a tube for centrifugation. **C)** Aspiration of the MSC-enriched stromal vascular fraction. **D)** Grafting of the stromal vascular fraction into the wound bed.

During the 10th treatment cycle (90 days after initiation), bilateral thigh liposuction was performed. The harvested adipose tissue was processed by centrifugation to obtain an MSC-enriched stromal vascular fraction. Application was performed after obtaining written informed consent from the patient, as part of an adjunctive clinical procedure. The technique was carried out during a supervised hands-on professional training activity in the institution to demonstrate clinical applicability. While not an essential step for the observed healing process, it was undertaken due to its potential local regenerative benefit and absence of significant additional risk. The procedure was not part of a research protocol and received no institutional or external funding (Figure 5).

**Figure 6:**
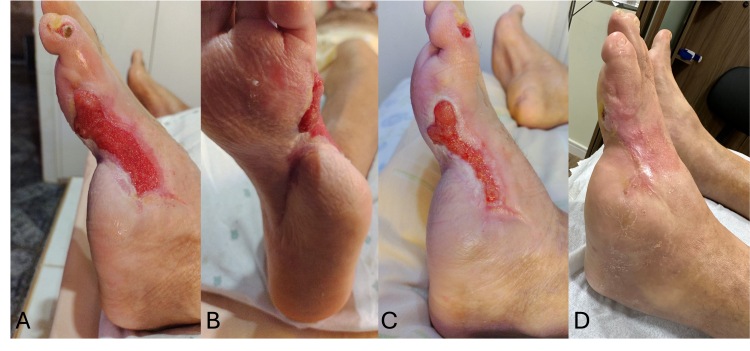
Photographs of the wound during the final phase of healing. **A** and **B)** Four months after the first intervention. **C)** After 5 months. **D)** After 7 months.

During outpatient follow-up in the day-hospital setting, the patient demonstrated progressive reduction of ulcer area, filling of the defect with granulation tissue, and peripheral epithelialization. Neuropathic pain was controlled with pregabalin 75 mg/day in addition to standard analgesics. Glycemic control was optimized with intensive insulin therapy (NPH and regular insulin). The wound showed a reduction of more than 90% of its initial area after 3 months of treatment, with complete epithelialization being achieved at 7 months, without signs of infection or recurrence, as demonstrated in Figure 6, which illustrates the final treatment outcome.

## DISCUSSION

Diabetic foot ulcers remain one of the most significant therapeutic challenges in vascular and endocrine practice worldwide, with substantial impact on patient morbidity and mortality as well as on public health care costs.^13,14^ The present case highlights the complexity of managing infected neuroischemic wounds, especially when associated with risk factors such as advanced age, mild renal insufficiency, severe infection, and chronic limb-threatening ischemia.

The coexistence of peripheral neuropathy, peripheral arterial disease, and infection constitutes the pathophysiological triad of diabetic foot ulcers, increasing the risks of amputation and mortality.^2,15^ Endovascular revascularization was pivotal in promoting wound healing and reducing the risk of major amputation, and infrapatellar angioplasty was the preferred technique due to its lower morbidity and the feasibility of repeat intervention.^3,15^

Early implementation of NPWT facilitated optimization of the wound bed, exudate control, and stimulation of granulation tissue formation. Studies have demonstrated that NPWT reduces healing time and infection rates, although its definitive impact on major amputation rates remains uncertain.^5,8,16^

The introduction of an ovine-derived ECM enhanced tissue remodeling by serving as a bioactive scaffold that promotes angiogenesis, cellular proliferation, and collagen deposition.^7,17,18^ The effectiveness of this material in chronic wound management has been demonstrated in controlled clinical studies.^13,19^

Autologous MSC therapy represents a promising frontier in the management of complex ischemic ulcers. MSCs promote the modulation of inflammation, stimulate angiogenesis and neocollagenesis, and exert local immunomodulatory effects.^20,21^ Recent meta-analyses have shown significant improvements in wound healing and tissue perfusion with this approach.^4,21^

The present case illustrates that the sequential integration of regenerative therapies with conventional techniques, such as revascularization and infection control, may contribute to limb preservation and complete wound healing, even in challenging clinical scenarios.

## CONCLUSION

The management of complex diabetic foot wounds, particularly those classified as infected neuroischemic lesions, requires a multimodal and individualized approach. In the reported case, the combination of endovascular revascularization, strict infection control, NPWT, ECM application, and MSC therapy resulted in complete wound healing and limb preservation. While it is not possible to attribute the favorable outcome to a single intervention, this case underscores the potential role of regenerative therapies as adjuvant components in multimodal treatment strategies.

## DISPONIBILIDADE DE DADOS

Dados não informados ou não utilizados: “Compartilhamento de dados não se aplica a este artigo, pois nenhum dado foi gerado ou analisado.”
