# Supplementary material for: Regenerative therapies for complex diabetic foot wounds: a therapeutic challenge
Source: J Vasc Bras. 2026 Apr 10;25:e20250071. doi: 10.1590/1677-5449.202500712 (PMC13142167; doi:10.1590/1677-5449.202500712)
Supplement: Translation

# Translation: Terapias regenerativas para feridas complexas do pé diabético: desafio terapêutico

**DOI:** 10.1590/1677-5449.202500711

**Authors:** Vinicius Tadeu Ramos da Silva Grillo, Eduardo Aparecido Romio, Caíque Damasceno Sousa, Gabriel Gomes Ranite, William Rodrigues da Silva, Pedro Luciano Melluci, Francisco José de Oliveira, Matheus Bertanha

**Affiliations:** 1 1 Universidade Estadual Paulista – UNESP, Faculdade de Medicina de Botucatu – FMB, Botucatu, SP, Brasil.; 2 Centro Universitário São Lucas – UNISL, Porto Velho, RO, Brasil.; 3 Instituto Vascular e Endovascular de Rondônia – IVER, Porto Velho, RO, Brasil.

**Keywords:** pé diabético, cicatrização de feridas, tratamento de ferimentos com pressão negativa, matriz extracelular, células-tronco mesenquimais, isquemia crônica crítica de membro

## Abstract

As úlceras do pé diabético representam uma das principais causas de morbidade, hospitalização e amputação não traumática de membros inferiores em pacientes com diabetes melito. O manejo eficaz dessas lesões requer abordagem multidisciplinar e terapias combinadas que contemplem controle infeccioso, otimização da perfusão e estímulo à regeneração tecidual. Este relato descreve o caso de um paciente com ferida neuroisquêmica grave e infectada, tratado com estratégia sequencial integrada: revascularização endovascular infrapatelar, antibioticoterapia direcionada, desbridamentos seriados, terapia por pressão negativa, aplicação de matriz extracelular de origem ovina e enxerto autólogo de células-tronco mesenquimais derivadas de tecido adiposo. O tratamento resultou em cicatrização completa da lesão e preservação do membro, sem recidiva após 7 meses de seguimento. Este caso destaca o potencial das terapias regenerativas avançadas no tratamento de feridas complexas e refratárias, ampliando perspectivas no cuidado de pacientes com pé diabético.

## INTRODUÇÃO

O diabetes melito representa uma das principais causas de morbidade e mortalidade no mundo, sendo o Brasil o sexto país em prevalência global, com aproximadamente 16,6 milhões de adultos diagnosticados, conforme o IDF Diabetes Atlas, 11ª edição, de 2025^1^ . Entre suas complicações mais graves e dispendiosas, destaca-se o pé diabético, resultante da interação entre alterações anatômicas, ortopédicas, vasculares, neurológicas e infecciosas^2,3^ . Essa condição compromete significativamente a qualidade de vida dos pacientes e é a principal responsável por amputações não traumáticas de membros inferiores^4^ .

As feridas complexas em pé diabético podem ser classificadas em neuropáticas, isquêmicas ou neuroisquêmicas, sendo estas últimas associadas a um pior prognóstico. Diversas opções terapêuticas têm sido desenvolvidas para o manejo dessas lesões, incluindo curativos avançados, desbridamento seriado, enxertos autólogos e heterólogos, terapia por pressão negativa (TPN), terapia celular com células-tronco mesenquimais (CTMs), terapia gênica, oxigenoterapia hiperbárica, substitutos dérmicos e antibióticos tópicos^5^ . Essas intervenções são consideradas terapias adjuvantes, sem impacto direto na fisiopatologia da doença, e ainda não foram incorporadas às diretrizes internacionais, que recomendam cautela devido à necessidade de estudos com maior amostragem e melhor qualidade metodológica^6^ . Além disso, o uso rotineiro de antibióticos tópicos não é recomendado nas úlceras do pé diabético.

Apesar das alternativas terapêuticas disponíveis, o tratamento das úlceras neuroisquêmicas ainda representa um desafio, devido à dificuldade de cicatrização, ao risco de infecção recorrente e à alta probabilidade de amputação maior. Estratégias multimodais associando revascularização, controle infeccioso rigoroso e terapias regenerativas emergem como alternativas promissoras no cenário atual^7,8^ .

O presente relato tem como objetivo descrever a aplicação integrada de terapias regenerativas em um paciente com lesão isquêmica complexa de pé diabético, ressaltando os aspectos desafiadores do caso e discutindo as perspectivas da medicina regenerativa no tratamento dessas lesões.

Este estudo foi avaliado e aprovado pelo Comitê de Ética em Pesquisa (CAAE 86657025.5.0000.0013, parecer nº 7.476.797).

## PARTE I – SITUAÇÃO CLÍNICA

Paciente do sexo masculino, 65 anos, deu entrada no pronto-socorro com história de ter pisado em brasa há 40 dias, evoluindo com lesões ulceradas em ambos os pés. Portador de diabetes melito tipo 2 e hipertensão arterial sistêmica há mais de 15 anos, fazia uso irregular de hipoglicemiantes orais e anti-hipertensivos, abandonados por conta própria nos meses anteriores. Relatava etilismo crônico e ex-tabagismo há mais de 30 anos. Sem histórico de cirurgias prévias.

Ao exame físico, apresentava necrose infectada do quarto e quinto pododáctilos do pé esquerdo, com linfangite ascendente no dorso do pé. No pé direito, observava-se pequena lesão superficial plantar, sem sinais flogísticos. Pulsos tibial anterior e pedioso eram palpáveis à direita; à esquerda, apenas o pulso poplíteo era detectável, sem pulsos distais e com índice tornozelo-braquial de 0,5. Não houve realização do teste de monofilamento, porém a anamnese e o exame físico indicavam neuropatia periférica simétrica distal.

A ferida foi classificada como grau 4 pela classificação de Wagner^9^ . Segundo a classificação WIfI da Society for Vascular Surgery, a lesão foi graduada como W3 I2 fI2, indicando ferida extensa (W3), isquemia moderada (I2) e infecção moderada (fI2), sugerindo alto risco de amputação sem intervenção adequada^10^ .

Não foram realizadas angiotomografia e arteriografia pré-operatórias devido ao quadro infeccioso sistêmico e à necessidade de intervenção cirúrgica urgente.

Os exames laboratoriais revelaram hemoglobina de 7,1 g/dL, leucocitose (19.900/µL), velocidade de hemossedimentação de 100 mm/h, proteína C reativa > 160 mg/L, albumina de 2,8 g/dL, creatinina de 1,7 mg/dL, sódio de 128 mEq/L e razão normalizada internacional de 1,47.

Foi iniciada antibioticoterapia empírica com ciprofloxacino (400 mg intravenoso [IV] 12/12h) e clindamicina (600 mg IV 8/8h), escolhidos por sua ampla cobertura para germes Gram-negativos e anaeróbios, frequentemente isolados em infecções polimicrobianas de pé diabético, além de hemotransfusão com três concentrados de hemácias. Realizou-se abordagem cirúrgica de urgência, com amputação do quarto e quinto artelhos esquerdos, fasciotomia plantar ampla e coleta de material para cultura, que identificou *Escherichia coli* multissensível. As imagens pré-operatórias e intraoperatórias iniciais encontram-se na Figura 1, que ilustra a extensão da necrose e o aspecto do leito da ferida após desbridamento inicial.

**Figura 1:** Fotografias da lesão. **A)** Avaliação inicial. **B)** Intraoperatório da primeira abordagem, após amputação do 5º metatarso. Nota-se necrose liquefativa do 4º metatarso. **C)** Aspecto final após a primeira abordagem cirúrgica, com amputação do 4º e 5º metatarsos, fasciotomia e desbridamento amplo.

## PARTE II – O QUE FOI FEITO

Após 4 dias da abordagem inicial, foi realizado novo desbridamento cirúrgico para retirada de tecido desvitalizado e otimização do leito da ferida, seguido de instalação da TPN, com pressão contínua de –125 mmHg (Figura 2). A escolha pela TPN foi baseada em julgamento clínico, sustentado por evidências que demonstram seu papel no controle do exsudato, na redução bacteriana e no estímulo à granulação tecidual^11,12^ .

**Figura 2:** Fotografias da lesão. **A)** Aspecto da lesão após a abordagem inicial. **B)** Curativo com terapia por pressão negativa (TPN). **C** e **D)** Evolução da lesão após início da TPN após 21 dias da abordagem inicial.

No 11º dia de internação, o paciente foi submetido a arteriografia diagnóstica, que confirmou a presença de lesões obstrutivas significativas nas artérias infrapatelares do membro inferior esquerdo: oclusão da artéria tibial posterior e múltiplas estenoses e suboclusões das artérias fibular e tibial anterior. Realizou-se angioplastia transluminal percutânea com balão das artérias infrapatelares, com sucesso técnico evidenciado pela recanalização e presença de pulso tibial anterior palpável ao término do procedimento, com índice tornozelo-braquial pós-procedimento de 0,9 (Figura 3). O manejo farmacológico subsequente incluiu dupla antiagregação plaquetária com ácido acetilsalicílico (100 mg/dia) e clopidogrel (75 mg/dia) por 90 dias, além do uso contínuo de estatina de alta intensidade (rosuvastatina 20 mg/dia).

**Figura 3:** Imagens de arteriografia por subtração digital do segmento infrapatelar. **A)** Arteriografia diagnóstica inicial com oclusão da artéria tibial posterior e múltiplas estenoses e suboclusões das artérias fibular e tibial anterior. **B)** Arteriografia final após angioplastia com balão das artérias fibular e tibial anterior. **C)** Arteriografia final com incidência anteroposterior do pé.

A antibioticoterapia foi mantida por 21 dias. Durante a internação, o paciente utilizou a TPN de forma contínua, com trocas realizadas a cada 7 a 10 dias, sempre acompanhadas de desbridamentos seriados em centro cirúrgico e registro da evolução do tecido de granulação. Após a alta hospitalar no 40º dia, o tratamento foi continuado em regime ambulatorial de hospital-dia. No total, foram realizados 11 ciclos de TPN, mantida ininterruptamente até o 120º dia, com trocas periódicas também em centro cirúrgico, sempre acompanhadas de desbridamento e reavaliação da ferida. Após esse período, o paciente seguiu em acompanhamento ambulatorial com curativos diários utilizando hidrogel, até a completa cicatrização da lesão.

A inclusão de matriz extracelular (MEC) derivada de submucosa de intestino delgado ovino (Endoform®) foi indicada devido à presença de tecido de granulação imaturo e bordas atróficas, visando estimular a angiogênese e a deposição de colágeno endógeno. A evolução foi monitorada por documentação fotográfica e mensuração periódica da área da ferida (Figura 4).

**Figura 4:** Fotografias da evolução da lesão. **A** e **B)** Após 40 dias da abordagem inicial. **C)** Após 60 dias da abordagem inicial, nota-se a colocação de matriz dérmica no leito do ferimento.

No 10º ciclo de tratamento (90 dias após o início), realizou-se lipoaspiração bilateral nas coxas. O tecido adiposo coletado foi processado por centrifugação para obtenção da fração estromal vascular rica em CTMs. A aplicação foi realizada após consentimento livre e esclarecido do paciente, no contexto de um procedimento assistencial complementar. A técnica foi executada durante atividade prática de capacitação profissional realizada no serviço, com o objetivo de demonstrar sua aplicabilidade clínica. Embora não fosse uma etapa necessária para a cicatrização observada, optou-se por sua realização por potencial benefício regenerativo local e ausência de riscos significativos adicionais. O procedimento não integrou protocolo de pesquisa e não recebeu qualquer financiamento institucional ou externo (Figura 5).

**Figura 5:** Fotografias do procedimento de terapia celular com enxerto de células-tronco mesenquimais realizado após 3 meses da abordagem inicial. **A)** Lipoaspiração do tecido adiposo da coxa. **B)** Transferência do material coletado para tubo para centrifugação. **C)** Aspiração da fração estromal vascular rica em células-tronco mesenquimais. **D)** Enxertia da fração estromal no leito da ferida.

Durante o tratamento ambulatorial no hospital-dia, o paciente evoluiu com redução progressiva da área ulcerada, preenchimento do defeito com tecido de granulação e epitelização periférica. A dor neuropática foi controlada com pregabalina 75 mg/dia, além de analgésicos comuns. O controle glicêmico foi otimizado com insulinoterapia intensiva (insulina NPH e regular). A ferida apresentou redução superior a 90% da área inicial após 3 meses de tratamento, com epitelização completa aos 7 meses, sem sinais de infecção ou recidiva, conforme evidenciado na Figura 6, que ilustra a evolução final do tratamento.

**Figura 6:** Fotografias da lesão durante a fase final de cicatrização. **A** e **B)** Após 4 meses da abordagem inicial. **C)** Após 5 meses. **D)** Após 7 meses.

## DISCUSSÃO

As úlceras do pé diabético continuam sendo um dos principais desafios terapêuticos da prática vascular e endocrinológica mundial, com impacto substancial na morbimortalidade dos pacientes e nos custos de saúde pública^13,14^ . O caso aqui relatado ilustra a complexidade do manejo de feridas neuroisquêmicas infectadas, especialmente quando associadas a fatores de risco como idade avançada, insuficiência renal leve, infecção grave e isquemia crônica crítica de membro.

A presença concomitante de neuropatia periférica, doença arterial periférica e infecção constitui o tripé fisiopatológico das úlceras de pé diabético, elevando o risco de amputação e mortalidade^2,15^ . A revascularização endovascular foi essencial para viabilizar a cicatrização e reduzir o risco de amputação maior, sendo a angioplastia infrapatelar a técnica de escolha devido à sua menor morbidade e possibilidade de repetição^3,15^ .

A utilização precoce da TPN permitiu a otimização do leito da ferida, o controle do exsudato e o estímulo à formação de tecido de granulação. Estudos mostram que a TPN reduz o tempo de cicatrização e infecção, mesmo sem impacto definitivo nas taxas de amputação maior^5,8,16^ .

A introdução de MEC ovina potencializou a reorganização tecidual, atuando como arcabouço bioativo que estimula angiogênese, proliferação celular e deposição de colágeno^7,17,18^ . A eficácia desse material em feridas crônicas já foi demonstrada em estudos clínicos controlados^13,19^ .

A terapia celular com CTMs autólogas representa uma fronteira promissora no manejo de úlceras isquêmicas complexas. As CTMs promovem modulação da inflamação, estímulo à angiogênese e neocolagênese, além de efeitos imunomodulatórios locais^20,21^ . Metanálises recentes evidenciam melhora significativa na taxa de cicatrização e perfusão tecidual com essa abordagem^4,21^ .

O presente caso exemplifica que a integração sequencial de terapias regenerativas com técnicas convencionais, como revascularização e controle infeccioso, pode contribuir para a preservação do membro e cicatrização completa, mesmo em cenários clínicos desafiadores.

## CONCLUSÃO

O tratamento de feridas complexas do pé diabético, especialmente aquelas classificadas como neuroisquêmicas e infectadas, exige abordagem multimodal e individualizada. O caso relatado demonstra que a combinação de revascularização endovascular, controle rigoroso da infecção, uso de TPN, aplicação de MEC e terapia celular com CTMs resultou em cicatrização completa e preservação do membro. Embora não seja possível atribuir o desfecho favorável a uma intervenção específica, o caso reforça o potencial das terapias regenerativas como adjuvantes em estratégias multimodais de tratamento.

## Data Availability

Data not applicable or not used: “Data sharing is not applicable to this article as no new data were generated or analyzed.”
